# Vibrational Branching Ratios and Asymmetry Parameters in the Photoionization of CO_2_ in the Region Between 650 Å and 840 Å

**DOI:** 10.6028/jres.106.038

**Published:** 2001-10-01

**Authors:** A. C. Parr, J. B. West, M. R. F. King, K. Ueda, P. M. Dehmer, J. L. Dehmer

**Affiliations:** National Institute of Standards and Technology, Gaithersburg, MD 20899-8440, USA; Daresbury Laboratory, Warrington, WA4 4AD, UK; Research Institute for Scientific Measurements, Tohoku University, Sendai 980, Japan; Office of Basic Energy Science, U.S. Department of Energy, Germantown, MD 20874, USA; National Science Foundation, 4201 Wilson Blvd., Arlington, VA 22230, USA

**Keywords:** asymmetry, autoionization, branching ratios, parameters, photoelectron spectra

## Abstract

The vibrational branching ratios and asymmetry parameters for CO_2_ have been determined in the wavelength region of 650 Å to near the ionization onset at about 840 Å. The study was performed using synchrotron radiation from the Daresbury storage ring that was dispersed with a 5 m grating monochomator that afforded resolution of 0.1 Å to 0.2 Å. This resolution allowed the study of the branching ratios and asymmetry parameters with enough detail to see the changes in the parameters within the pronounced autoionization structure in CO_2_ in this wavelength region. While the electron spectrometer resolution was not sufficient to resolve the spin orbit and Renner-Teller splitting in the photoelectron spectra, we are able to fit the data with a model that identifies the major structure in terms of the symmetric stretch and elements of the asymmetric stretch and bending modes. A calculation of the expected relative vibrational excitations based upon the Franck-Condon principle clearly showed non-Franck-Condon behavior in some of the vibrational-electronic transitions.

## 1. Introduction

The study of CO_2_ photoionization has historically attracted much attention due to the importance of the ionization of CO_2_ in the photophysics of planetary atmospheres, including the Earth’s atmosphere [1,2]. Additionally, CO_2_ is an integral part of the carbon cycle for plant life and, as a consequence, the photochemistry and photophysics of this molecule has been the subject of considerable interest by a number of scientists over the last several decades. The ready availability of high purity samples of CO_2_ and its ease of use in various experimental arrangements have produced many experimental efforts studying its various properties [3–7].

The ionization onset region in the photoabsorption of CO_2_ contains a number of strong autoionization features that converge on the A^2^∏_u_ and $B^{2}\sum_{u}^{+}$ states seen in a photoionization mass spectrometric study by McCulloh [8] who tabulated them and gave the various series identifying labels which we will use in this work. Hubin-Franskin et al. [9] and Holland and Hayes [10] have used photoionization techniques coupled with synchrotron radiation sources to study the branching ratios or vibrational transitions in the wavelength region reported in this study [9,10]. Baer and Guyon [11] used synchrotron radiation to study autoionization and isotope effects in the photoionization in the 620 Å to 900 Åwavelength region. These researchers used a threshold photoelectron spectrometer system and identified a number of vibrational transitions that are ordinarily weak or forbidden by selection rules.

Recently Buenker et al. [12] have performed calculations and reviewed the status of the studies on CO_2_ electron energy loss spectroscopy. This technique can give results for cross sections and oscillator strengths that are similar to those obtained by photoionization and can be a direct test of calculational techniques. Takeshita et al. [13] recently calculated the ionization energies and the Franck-Condon factors for CO_2_ photoionization. Johnson and Rostas [14] reviewed the spectroscopic literature for the vibronic structure of the ground state and first few excited electronic states of CO_2_. The spectroscopic results given by these authors were most useful in developing a method of analyzing the data reported in this work.

In a previous publication [15], we gave results for the branching ratios and asymmetry parameters for CO_2_ in the wavelength region of 685 Å to 795 Å for some of the autoionizing lines in the Tanaka-Ogawa (TO) series, the Henning sharp (s) and diffuse (d) series. However, we did not publish the extensive additional data we had obtained as a result of analyzing the photoelectron spectra taken in this wavelength region. The results reported here are a complete analysis of all our data, providing extensive information on the photoionization behavior of CO_2_ in this wavelength region. Another publication from our collaboration used the angular distribution information to determine the electronic structure, symmetry, and decay dynamics for members of the TO series[16]. It was noted in these publications, as well as in preliminary work done in the early 1980s, that the strength of the vibrational progressions were not described by a simple Franck-Condon model of photoionization [17]. We also explored the effects of vibronic coupling on the intensity distribution in the 4 σ_g_ photoionization channel of CO_2_ [18]. Hubin-Franskin et al. studied CO_2_ autoionization and pointed out that the use of the method worked out by Smith and modified by Eland to apply the Fano-Mies configuration interaction theory to molecules could be used to explain portions of the non-Franck-Condon behavior seen in the photoionization of CO_2_ [19–23]. These papers pointed out the difficulty of completely accounting for the vibrational-electronic transition intensities due to configuration mixing of electronic states, vibronic and rotational interactions, and the competition between the many paths available for an excited state neutral molecule to decay. The Smith formalism requires a knowledge of the Franck-Condon factors for all the transitions involved, ground state to autoionizing level and autoionizing level to all the possible final states. While this calculation is possible with diatomic molecules, it is much more difficult with larger molecules with additional degrees of freedom. In the case of CO_2_, the situation is even more complicated due to Renner-Teller splitting of the electronic levels when the bending mode is excited. As a result, there has been no systematic theoretical exploration of the effects of autoionization on the vibrational branching ratios and asymmetry parameters for CO_2_.

In our previous publication concerning the branching ratios and asymmetry parameters in this wavelength region we presented only a small subset of the data we have on CO_2_ in this wavelength region. We present here a detailed explanation of the data accumulation techniques and a complete discussion of the analysis of the data that was not possible in the shorter summary of this work [15]. To be consistent with the earlier NIST publications on this topic, we have used the unit of the Angstrom to describe the wavelength scale used for these experiments even though this unit is no longer sanctioned as an acceptable metric system unit.

## 2. Experimental Procedure

**Fig. 1:**
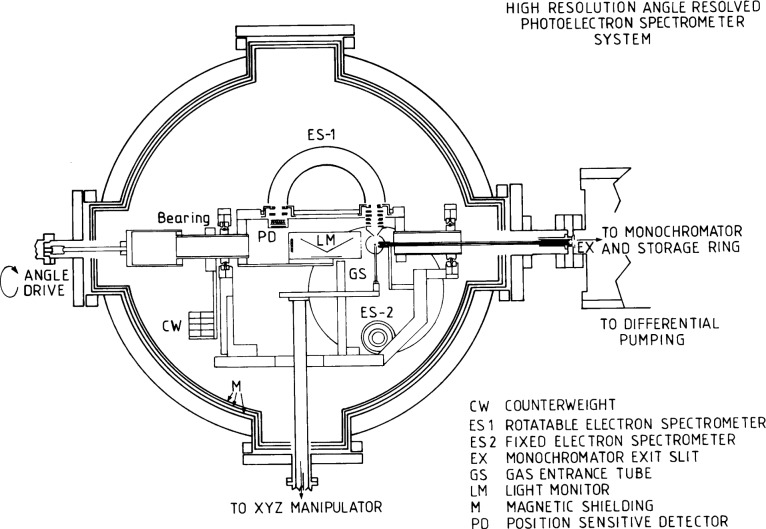
Cross section view of the electron spectrometer chamber. The chamber is 76 cm in diameter and is 91.4 cm in length. The major aspects of the instrument are labelled in the figure.

This experiment was carried out on the 5 m normal incidence monochromator fitted to a beamline at the Daresbury SRS, providing a photon flux of ≈10^10^ photons/s within a bandpass of 0.1 Å in the spectral region from 650 Å to 840 Å [24]. The light was brought into the experimental chamber by a 2 mm internal diameter glass capillary light guide whose capillary aperture was placed close to the exit slit of the monochromator. In addition to offering a low-loss transport for the vacuum ultraviolet radiation, the capillary also served to maintain a pressure differential between the experimental chamber and the ultra high vacuum of the optical monochromator. A schematic diagram of the experimental apparatus is shown in Fig. 1. The light guide extends from the exit slit, EX in Fig. 1, to the interaction region above the gas entrance tube, GS in Fig. 1, and can be as long as 30 cm dependent upon exact placement of the experimental chamber with respect to the monochromator.

The electron spectrometer system comprises two 100 mm mean radius hemispherical analysers, one rotatable about the incoming light beam as an axis and the other fixed, contained in a chamber shielded from magnetic fields by three layers of μ-metal. The radiation from the monochromator is polarized, its polarization depending upon the optics of the monochromator and the subtended angles of acceptance of the synchrotron radiation. In our configuration, the light is polarized with between 75 % and 80 % of the light intensity having its electric field vector ( $\vec{E}$-vector) perpendicular to the plane of the paper in Fig. 1. In this configuration, the fixed analyser accepts electrons ejected parallel to the $\vec{E}$-vector of the incident radiation. The fixed analyser is ES-2 in Fig. 1. The other electron spectrometer is rotatable about an axis defined by the direction of the light and hence collects electron in the plane of the $\vec{E}$-vector. This movement allows the angular distribution of the photoelectrons to be explored completely and is sufficient to determine the angular asymmetry parameter for the scattering process. The entrance lenses for the spectrometers are three element zoom lenses based upon the design of Harting and Read [25]. The entrance cone to the lens system has a small aperture, usually about 1 mm in our experiments, which acts as the limiting aperture for determining both the energy and angular resolution of the system. The zoom lens focuses the electrons from a small interaction volume determined by the size of the light beam exiting the capillary and the size of the gas jet exiting the gas entrance tube onto the entrance plane of the hemispheres. The pass energy of the electron analyzer and focus voltages are set by external controls. The pass energy remains fixed for a particular set of experiments, and the other voltages are appropriately varied to scan the electron energy spectrum as required by using an automated data control system. The electrons are dispersed upon passing through the analyzer hemispheres and focussed on the hemisphere exit plane. Since the apparatus was first described in the literature, the electron spectrometers have been fitted with position sensitive detectors which are placed near the exit focal plane of the hemispheres [26]. This allows for the simultaneous detection of a range of energies in the photoelectron spectra and thereby improves the data quality for a given period of data accumulation, compared to using a conventional electron multiplier behind an exit slit.

The polarization of the incoming light was measured using a three mirror polarizer with tungsten mesh and plate photodiodes which could be rotated with the rotatable analyzer through 90° in order to determine the light polarization. The polarization detection device was constructed based upon considerations given by Horton et al. [27]. The polarization was checked frequently since small movements in the storage ring beam position, and the mirrors focussing the light onto the entrance slit of the monochromator, can have a marked effect on the polarization. It was found that, provided the pre-mirror adjustments were kept optimized for maximum photon flux at the exit slit of the monochromator, the polarization would remain stable during the accumulation of a particular data set. The tungsten wire mesh at the entrance of the polarizer served as a photocathode for monitoring the intensity of the incoming light beam. A tungsten plate serving as a second photocathode collected the beam remaining after three reflections of the beam. The ratios of these photo-currents as a function of the angular position of the movable electron spectrometer provided the data necessary to determine the polarization of the light.

Calibration of the energy response of the analyzers was performed using the known values of the cross section and asymmetry parameters for argon or helium gas and following standard procedures outlined in the literature [28–30]. For all the spectra reported here the electron spectrometer resolution was determined from the rare gas calibration to be 41 meV for the fixed analyser and 46 meV for the rotatable analyzer. The 5 m monochromator resolution was 0.1 Å(≈2 meV) for the measurements taken at wavelengths shorter than 750 Å. At wavelengths longer than this, where the structure in the absorption spectrum is less dense, the resolution requirements could be relaxed and a wavelength resolution of 0.2 Å was used. The data were accumulated by simultaneously taking photoelectron spectra at two angles with respect to the polarization direction by utilizing both electron spectrometer systems. Since the two analyzers could be positioned at different angles, a particular data point did not require rotation of the movable electron spectrometer system. The wavelength on the monochromator was then incremented by 0.1 Å and another set of photoelectron spectra taken. The light polarization was checked periodically by a 90° rotation of the movable electron spectrometer. During data accumulation, the time spent at a particular electron kinetic energy was determined by integration of the light flux signal to some predetermined amount so that all the points in a particular data set would be correctly normalized to the same total light flux.

The differential cross section for photoabsorption in the dipole approximation for a randomly oriented gas may be expressed as

$$\frac{d\sigma_{\nu }}{d\Omega }=\frac{\sigma_{\nu }}{4\pi }[1+\frac{\beta_{\nu }}{4}\left(3P\;\cos\;2\theta +1\right)$$ (1)

where *θ* is the angle between the major polarization axis and the ejected electron, *P* is the degree of polarization of the incoming light, *Ω* is the solid angle of collection of the photoelectrons, and *σ_ν_* is the partial cross section for the vibrational-electronic channel corresponding to the photoelectron being detected. The total cross section for a particular electronic channel is the sum of the partial cross sections of the individually resolved vibrational channels. The vibrational branching ratio is defined as the partial cross section for that channel divided by the total cross section for the electronic state. The number of photoelectrons per steradian per unit light flux, d*N_ν_*/d*Ω* is proportional to the differential cross section and hence we can recast the above equation into one that refers to the measurable parameters of the experiment:

$$\frac{dN_{\nu }}{d\Omega }=\frac{N_{\nu }}{4\pi }[1+\frac{\beta_{\nu }}{4}\left(3P\;\cos\;2\theta +1\right)$$ (2)

*N_ν_* is the total number of electrons detected in a particular vibrational channel summed over the all solid angles. Measurements were made simultaneously at *θ* = 0° and *θ* = 90° and hence *β_ν_* and *N_ν_* could be directly deduced from the two spectra [31]. The branching ratio for a particular transition is the ratio of *N_ν_* with respect to the sum of all the *N_ν_*’s for a particular electronic excitation. The data reported here were taken only for transitions that left the ${\text{CO}}_{2}^{+}$ molecule in the X ^2^∏_g_ ground electronic state. Altogether about 1500 data sets were taken in the wavelength region 650 Å to 890 Å.

**Fig. 2:**
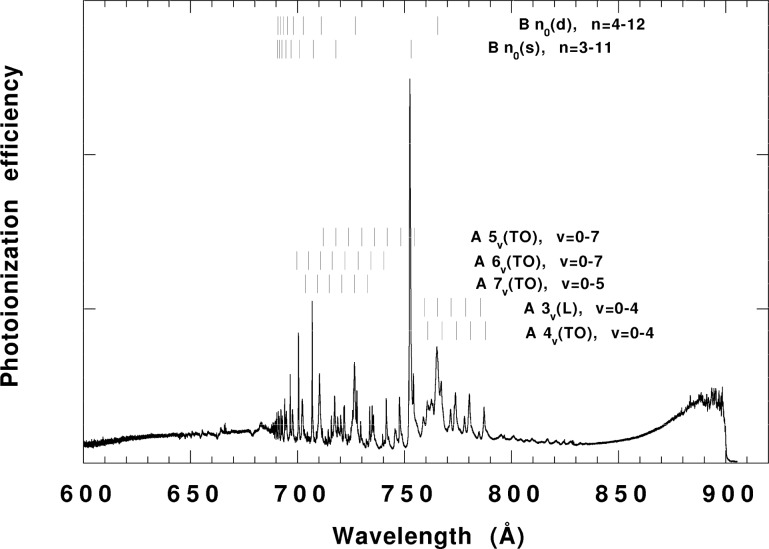
Photoionization efficiency spectrum for CO_2_ in the wavelength region from ionization onset to 600 Å,. The data were taken using a photoionization mass spectrometer system with a wavelength resolution of 0.12 Å.

Figure 2 shows the photoionization efficiency (relative photoionization cross section) for ${\text{CO}}_{2}^{+}$ in the wavelength region of interest in the present study. The data were taken using a rotationally cooled sample of CO_2_ and a laboratory light source coupled to a quadrupole mass filter [16,32]. A 1 meter near-normal incidence monochromator provided dispersed radiation with a wavelength resolution of 0.12 Å. This rotationally-cooled spectrum shows more detailed and sharper structure, particularly near ionization onset, than does the spectrum obtained by Berkowitz [33] with a slightly better wavelength resolution of 0.07 Å. The spectrum shows members of the TO series that have as limits the A state of ${\text{CO}}_{2}^{+}$ vibrational levels. The notation A*n_ν_* (TO) *n* = 4,5, .. etc., means the level is a member of the TO series having the *n_ν_* vibrational level of the A state of ${\text{CO}}_{2}^{+}$ as its limit. The symbol B*n*_0_(s,d) *n* = 3,4,” etc. is a Rydberg level of quantum number n having as its series limit the B state of ${\text{CO}}_{2}^{+}$ in the vibrational ground state. The s or d refers to the Hennings sharp or diffuse series. The notation A3*_ν_* (L) *v* = 0, .. refers to a level of the Lindholm series with principal quantum number 3 that has the A state as its limit and has a vibrational excitation of *v* in the symmetric stretch mode.

## 3. Analysis of the Data

The analysis of the large amount of data taken on CO_2_ required careful organization of the data and the development of a reliable method of extracting the highly detailed information on vibrationally resolved asymmetry parameters and branching ratios that the data contained. The raw data must first be corrected for the differing detection efficiencies of the two electron energy analyzers. This correction is a function of the kinetic energy of the electron and is accounted for by the determination of an energy dependent correction factor from angular distribution data measured by the two electron spectrometers for rare gases as discussed in the previous section. The correction function was frequently checked during the course of the experiment by measuring the photoelectron spectra of a rare gas as a function of wavelength over a wavelength range sufficient to generate photoelectrons with kinetic energies equivalent to those produced in the photoionization of CO_2_. The system exhibited excellent stability in this regard as, once the lens voltages and other operational parameters of the electron spectrometers were set, the relative efficiencies of the analyzers did not vary in any significant manner.

The ^2^∏_g_ ground state of ${\text{CO}}_{2}^{+}$ is spin-orbit split into two levels that are 19 meV apart and the bending mode populated in the resonance regions is split a similar amount by Renner-Teller coupling. This structure was not resolved and hence the effect of these interactions was to broaden the spectral peaks observed in the ${\text{CO}}_{2}^{+}$ photoelectron spectra. This could be accounted for by examining the shape of the photoelectron peaks seen in the rare gas spectra and appropriately adding two such peaks that are offset by the 19 meV splitting causing the broadening. Using this procedure and some testing on fitting some actual ${\text{CO}}_{2}^{+}$ spectra resulted in obtaining 47 meV and 56 meV as the best effective resolutions of the two analyzers to be used in the fitting routine. The fitting routine allowed for the resolution to be a free parameter or a constrained parameter but the best overall fittings were obtained when the resolutions were fixed at these values and not allowed to vary during the fitting process. The fitting routine used as a model a series of peaks whose widths were fixed and whose relative positions were fixed by the spectroscopic positions of the vibrational energy levels. The fitting routine was a nonlinear least squares analysis [34] that was customized at NIST for the type of spectra observed.

The spectroscopic values of the vibrational parameters were taken from the literature [35,36] and fitted to our data using *ω*_1_ = 156.7 meV for the symmetric stretch, *ω*_2_ = 0.060 meV for the bending mode, and *ω*_3_ = 182.3 meV for the asymmetric stretch. These values gave good fits to the data over a broad range of electron kinetic energies and represent an averaging over the levels split by the Renner-Teller interaction. The transitions to the asymmetric stretch and bending mode with odd number of quanta are forbidden by selection rules from the ground state of the neutral molecule but have been observed in other photoelectron work reported in the literature [37]. Though these transitions are not allowed by the Franck-Condon principle, their existence is well established experimentally and can be attributed to anisotropic interactions of the escaping electron with the molecular core. The presence of these transitions and the Renner-Teller splitting of the levels gives rise to an overlapping of the possible transitions, and this makes unique identification of the photoelectron peaks we observe impossible except in some prominent circumstances. Chambaud, Gabriel, Rosmus, and Rostas have identified many of the low lying levels of ${\text{CO}}_{2}^{+}$, providing a basis for estimating the positions and compositions of the levels we observe [38]. Using the notation (*v*_1_,v_2_, *v*_3_) where *v*_1_ is the quantum number of the symmetric stretch, *v*_2_ the quantum number of the bending mode, and *v*_3_ that of the asymmetric stretch, we find the spectral features can be adequately assigned to the following vibrational levels:

(*v*_1_,0,0) *v*_1_, = 0, 1…
(*v*_1_,1,0) *v*_1_, = 0, 1…
(*v*_1_,2,0) *v*_1_, = 0, 1, …4
(*v*_1_,0,1) *v*_1_, = 0, 1, …5
(*v*_1_,1,2) *v*_1_, ≥ 3

The upper value of *v*_1_ is determined by the span of the energy range covered by the photoelectron spectra. In some cases it was not necessary to include the (*v*_1_,0,1) or (*v*_1_,1,2) levels, as the intensity in the high binding region was small and the structure was adequately represented by the simpler set of levels. Table 1 gives the vibrational levels used in the fitting model in the energy range of one eV of vibrational excitation. The levels are identified as discussed above and the energy position is given with respect to the (0,0,0) vibrational ground state of ${\text{CO}}_{2}^{+}$.

It is possible other types of excitations may contribute to the spectra observed. The appearance of finite values for intensity at an energy determined by the above energy levels does not constitute an unambiguous identification of a particular vibrational level for ${\text{CO}}_{2}^{+}$. As an example, our data would not separate out the (2,0,0) components from those of components of the split (1,0,1) and the (1,2,0) levels. The (1,2,0) level is split into 4 levels by the Renner-Teller and spin-orbit interactions, the upper two of which are approximately coincident with the (2,0,0) levels. The electron energy resolution of the present experiment was not sufficient to warrant attempts at resolving the levels due to the splittings. Similarly, the intensity observed for “forbidden” transitions to levels of type (*n*,1,0) should not be regarded as definitive. In an earlier publication-where we studied CO_2_ photoionization with an electron spectrometer resolution of about 19 meV, members of the (*v*_1_,0,2) progression were clearly visible [16] which indicates that the asymmetric stretch excitations can play a role in the photoabsorption of CO_2_. In this lower resolution study, these excitations could not be resolved from those excitations shown in Table 1. Our goal in the fitting was to identify peak positions which would best account for the electron intensity observed and which could be identified with predicted vibrational structure.

To accomplish the fitting, relative peak positions were fixed by the spectroscopic model chosen, the peak widths were fixed as determined in the previous discussion and the heights of the photoelectron peaks were allowed to vary in order to minimize the fitting errors with the least squares technique. After many trials it soon became evident which vibrational modes were the most important to achieve a reasonable fit. This set of vibrational modes was adopted as a basis for the analysis. At energies 2 eV above the threshold, it was not possible to resolve the individual vibrational peaks and they were often seen as a modulated background whose intensity was accounted for by high members of the above progressions. In the wavelength region of 652.08 Å to 680.98 Å it was sufficient to use only the first three vibrational progressions shown above; in the region 689.7 Å to 713.9 Å it was necessary to use the first four levels; and in the remainder the complete set was used. The only exception to this was the use of levels of the sort (*v*_1_,1,1) in the 689.77 Å to 694.64 Å region where this addition seemed to improve the fit somewhat but did not suggest itself elsewhere and hence will not be pursued in this report.

**Fig. 3:**
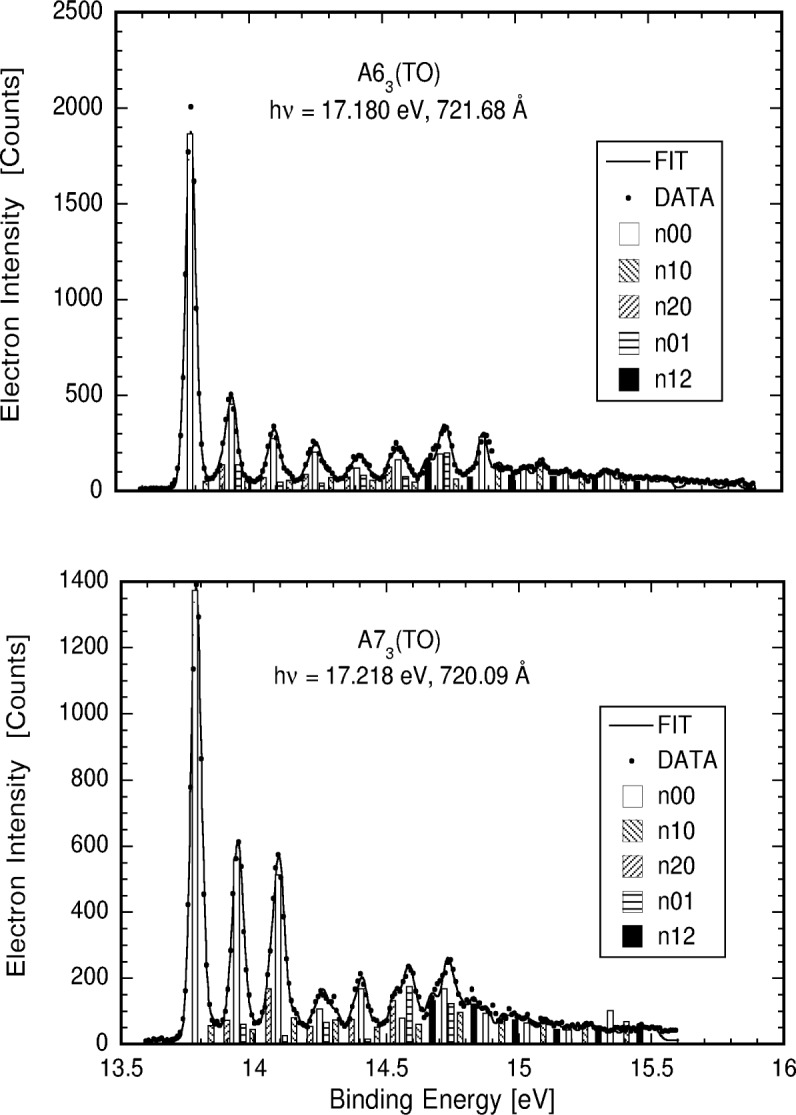
Two photoelectron spectra taken on the 0° analyzer at nearby wavelengths. The spectrum differ considerably but the same model fits both relatively well. The solid line is the fit and the dots are the actual data. The vertical columns represent the intensities of the various vibrational transitions as defined in the figure that contribute to the intensity at that point.

After fitting, each data set and the fit were inspected, and the model varied by adding or removing vibrational modes as required to achieve the best fit. Figure 3 shows the quality of the fit obtained by the 0 analyzer at a photon energy of 17.180 eV. The lower portion of Fig. 3 shows the fitting results for the same analyzer at a photon energy of 17.218 eV. Both fits were accomplished using the same model for vibrational state composition even though the structure is substantially different in the two closely spaced regions of the absorption spectrum. The vertical lines in the figures are the calculated amplitude of the contributions from the progression listed. The solid line, labelled FIT, represents the fit of the calculated spectrum to the raw data (DATA in the figure). The data points are for the most part obscured by the fitted line, an indication of the quality of the fit.

It can be seen that the data are of sufficient quality to give reliable intensities for the vibrational components marked, allowing vibrational branching ratios down to the 5 % level to be measured. In the figures shown later, the uncertainties include the statistical element (type A uncertainty) derived from the fitting procedure, and further contributions due to uncertainty in the polarization of the incoming light and the calibration of the tungsten photodiodes (type B uncertainty) [39]. In general, the statistical uncertainty gave the largest effect. The error bars shown on the data represent the combination in quadrature of the uncertainties of type A and type B propagated with the appropriate error propagation for the equations given earlier in this paper.

**Fig. 4:**
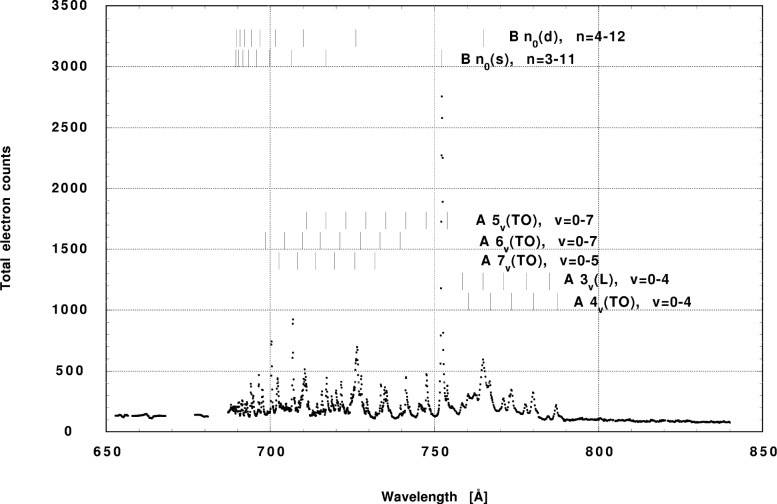
Total electron count measured with the electron spectrometer system plotted as a function of wavelength in Å. The main autoionizing Rydberg levels are identified as described in the text.

Having obtained the intensities for the vibrational levels for each of the two analyzers, we then used these data, together with the measured light polarization and the electron spectrometers’ energy efficiency curves, to calculate the asymmetry parameters and branching ratios. The total electron count as a function of wavelength is shown in Fig. 4. This was obtained by summing over the values of the calculated *N_ν_* for all the levels calculated for the X ground state. The intensities were corrected for the varying efficiency of the tungsten photodiode used to measure the light intensity and can be seen to agree closely with the results shown for the photoionization efficiency in Fig. 2 [40].

**Fig. 5:**
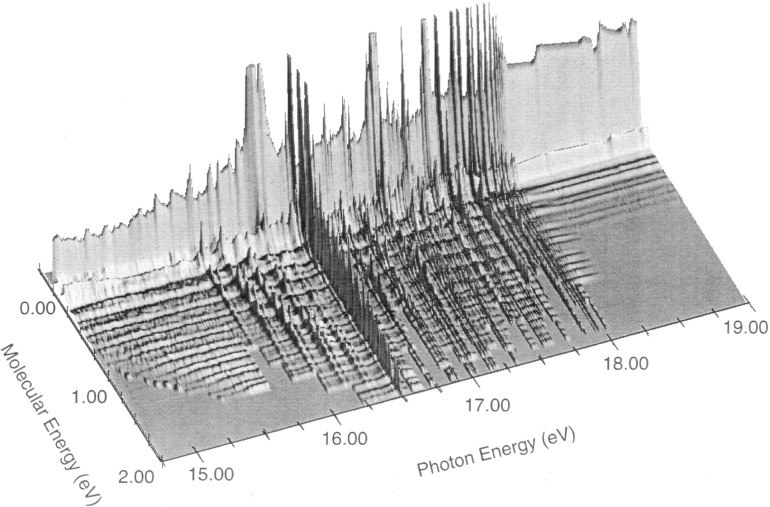
Three dimensional plot of the results of the fitting routine for all the spectra measured. The molecular energy axis represents the vibrational excitation of the ionic ground state of ${\text{CO}}_{2}^{+}$. The plot dramatically shows the increased vibrational excitation caused by autoionization phenomena.

The three dimensional plot shown in Fig. 5 shows the electron spectra where the most intense members have been truncated in order to reveal the detail in the higher vibrational modes. Examination of this figure reveals the amount of ${\text{CO}}_{2}^{+}$ vibrational excitation occurring as a result of autoionization which populate molecular energy levels well above 0.5 eV. This excess population of excited state ${\text{CO}}_{2}^{+}$ would have consequences on the reactivity of this molecule in atmospheres where ${\text{CO}}_{2}^{+}$ is formed by uv irradiation. When the number of electrons as a function of molecular energy are summed as shown in Fig. 5, the total electron count as shown in Fig. 4 is obtained.

## 4. Discussion

In Figs. 6–20, the spectra for *β* and branching ratio are shown, for all the vibrational levels for which there was significant intensity. These have been selected from the complete data set because they show structure identifiable with the resonance positions; the complete data set is available from a NIST database as described below.

To proceed with further analysis, we consider the Born-Oppenheimer approximation and the Franck-Condon (FC) principle. These two approximations have been used successfully for the analysis of vibrationally-resolved photoelectron spectra. Within these well-known approximations, the branching ratio in the photo-electron spectrum, for ionization directly to the continuum, is given by the FC factor between the ground level and the vibrational levels of the ionic state. The *β* values are expected to be independent of the vibrational quantum numbers of the ionic state. If photoelectron emission occurs through the resonance, on the other hand, one should take account of both the resonant and non-resonant components with the help of Fano’s resonance theory [43]. Then the branching ratio is governed not only by the FC factor between the ground level and the vibrational levels of the ionic state but also by the FC factor between the vibrational level of the excited state and the vibrational levels of the ionic state. If the resonant enhancement is so strong that the non-resonant component is negligible (i.e., |*q* | ≫ 1 in Fano’s resonance formula [43]), the branching ratio is dominated solely by the FC Factor between the vibrational level of the excited state and the vibrational levels of the ionic state. In this situation, the *β*-values are again independent of the vibrational quantum numbers of the ionic state.

In our previous publication [15], we made assumptions mentioned above and the approximation of neglecting the contribution from the non-resonant ionization on the basis that the line shapes were in general Lorentzian. In terms of the Fano [43] theory, this means that the |*q*|-value is very large, indicating that the continuum component is very small. Then the FC factors for the transition between the vibrational level of the resonant state and the vibrational levels of the final ionic state were calculated. The approximation was made that the potential parameters for the resonant state are the same as those for the ionic state to which the resonance converges. Clearly this approximation should improve the higher up the Rydberg series the resonance is, and some qualitative agreement with this method of calculation was found. For the (0,0,0) branching ratio, the A*n*_1_ and A*n*_2_ (*n* = 4–7) TO series are calculated to have a slowly increasing branching ratio as one proceeds up the Rydberg series approaching the theoretical value. However, there was poor agreement for the branching ratios as one proceeds along the members of the main vibrational progression (*n_ν_*,0,0), as noted in [15], and it was clear that considerable intensity moves from this progression into other vibrational levels.

To take account of the intensity transfer from the main progression to the other vibrational modes as a first approximation, we have added the intensities of the nearest vibrational member above and below the (*v*,0,0; *v* = 1–7) vibrational members for the same resonances as in the earlier paper. For example, referring to Table 1, we added the values for (0,2,0) and (0,0,1) to the branching ratio for (1,0,0) and similarly for the other vibrational levels up to (5,0,0). Our labelling of the nearest levels changes for (6,0,0) and (7,0,0), but, as pointed out earlier, this labelling is not definitive given the resolution of our experiment. It was derived from the fitting process, but it would be impossible to confirm that intensity found in the (3,1,2) level could not in fact be attributed to the (5,2,0) level which was not included in our model. In the case of the (6,0,0) and (7,0,0) levels we added in the intensities from the vibrational levels just above and below as with the other vibrational levels.

The results are shown in Fig. 21 as a bar chart, where each group of intensities corresponds to the resonance shown. The heights of the bars correspond to the branching ratios for (0,0,0) to (7,0,0), going from left to right in each group, with the additional contributions added as described above. In Fig. 22, the theoretical values from the previous work are shown. In principle these should be the same for any given Rydberg series, and are labelled accordingly. Some similarities are immediately obvious:

- For the first set of resonances, A*n*_0_, n = 4 – 7, although theory puts (1,0,0) more intense than (0,0,0), the intensity dies away as one proceeds up the vibrational progression much as theory suggests.
- For the next set, A*n*_1_, n = 4 – 7, a minimum at (2,0,0) and a maximum at (5,0,0) is more or less reproduced. For A7_1_, the (0,0,0) intensity is about right, and here the theory looks quite good.
- For the next set, A*n*_2_, n = 4 – 7, a low value of (1,0,0) is not reproduced, but the minimum at (3,0,0) is there, together with higher values of (5,0,0), (6,0,0) and (7,0,0) compared with the other resonances. A5_2_, also looks particularly good; (0,0,0) is a bit low, but the overall structure is clearly there.
- In the last set, A*n*_3_, n = 4 – 7, two minima are predicted at (2,0,0) and (5,0,0). This fits well with A5_3_, and A*7*_3_, with some suggestion of it at A4_3_. Again theory puts (1,0,0) as most intense, which is not the case.
- The general trend for intensity to move to higher vibrational members as one moves to higher vibrational excitations in the resonances is also reproduced. This is evident in Fig. 5; in the resonance regions vibrational structure extends, with considerable intensity, well into the regions of high vibrational energy, in contrast to the off-resonance spectra where almost all the intensity is concentrated in the first few members of the main vibrational progression.

It seems, therefore, that our FC model, which neglects direct ionization and takes account of the intensity transfer to the nearest different vibrational levels, can be applied in resonance regions for some cases. We do not at the present time have any general rules for quantifying the intensity transfer to the nearest vibrational levels for all the spectral regions. Some disagreements may be attributed to the neglect of direct ionization.

An analysis on the basis of that carried out by Smith [20] for O_2_, in which the Fano parameters are derived by fitting the experimental line shape to the Fano formula, includes the direct ionization component. Given our assumption above based on Lorentzian line shapes, and the fact that there are many overlapping lines, it was not thought worthwhile to follow this procedure here, although for the Hopfield series in the region between 680 Å and 720 Å where the line shapes are not symmetric it may be more appropriate. Even here, however, our experiment has insufficient resolution to separate overlapping structure clearly and values of the Fano parameters obtained by any fitting procedure are likely to be in some doubt.

Turning our attention to the measured *β*-values, we note that the average value of *β*, where we define “average” as an underlying value of *β* outside resonant structure over the whole wavelength range (i.e., the regions corresponding to direct ionization), is between 0 and –0.5 in general for all the spectra shown. Even for the angular distribution measurements not shown here, because there was considerable scatter in the data, this still appeared to be the case. This would be consistent with, though not evidence for, validity of the Born-Oppenheimer approximation and the FC principle for direct ionization. The fact that the *β* value is negative indicates the presence of “parity unfavoured” transitions [41]; an analysis on the basis of the angular momentum theory proved partially successful for the oxygen molecule [42], where only one vibrational mode is present. Given the larger number of decay channels available for CO_2_, an analysis on this basis is unlikely to be conclusive.

**Fig. 6:**
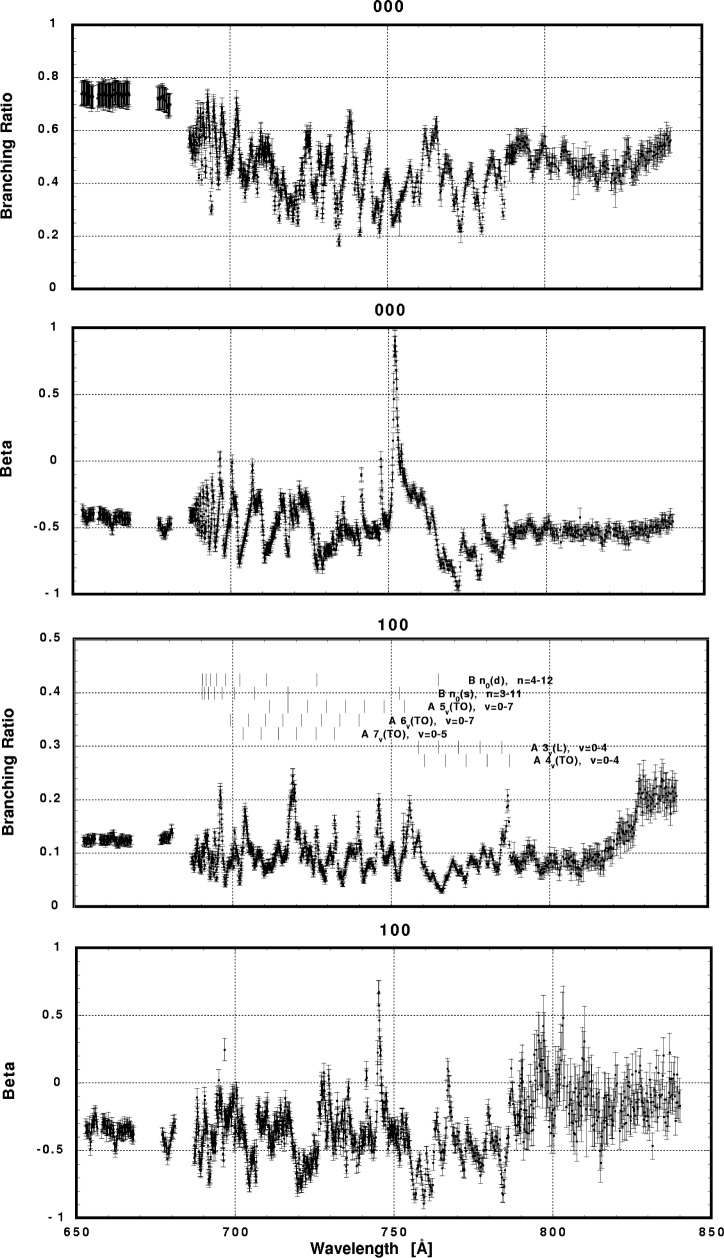
Plots of the asymmetry parameter and branching ratio for the (000) and (100) levels of the ${\text{CO}}_{2}^{+}$ ground state.

In the resonance regions, however, we notice breakdown of the FC model applied as described earlier, since that model dictates that the *β*-values should not change for vibrational levels within the same progression. Note for example in Fig. 6 the markedly different response of *β*, in the case of the two vibrational levels shown, to the B 3_0_(s) resonance at ≈751 Å. The breakdown of the simple picture may be just due to neglect of the non-resonant component in the region of the resonances. The B 3_0_(s) peak is however the strongest among all the peaks in the total cross section spectrum, and it is hard to believe that the non-resonant component can contribute to this dramatic effect. The different response of the different vibrational levels within the same progression suggests breakdown of the Born-Oppenheimer approximation and/or the FC principle.

**Fig. 9:**
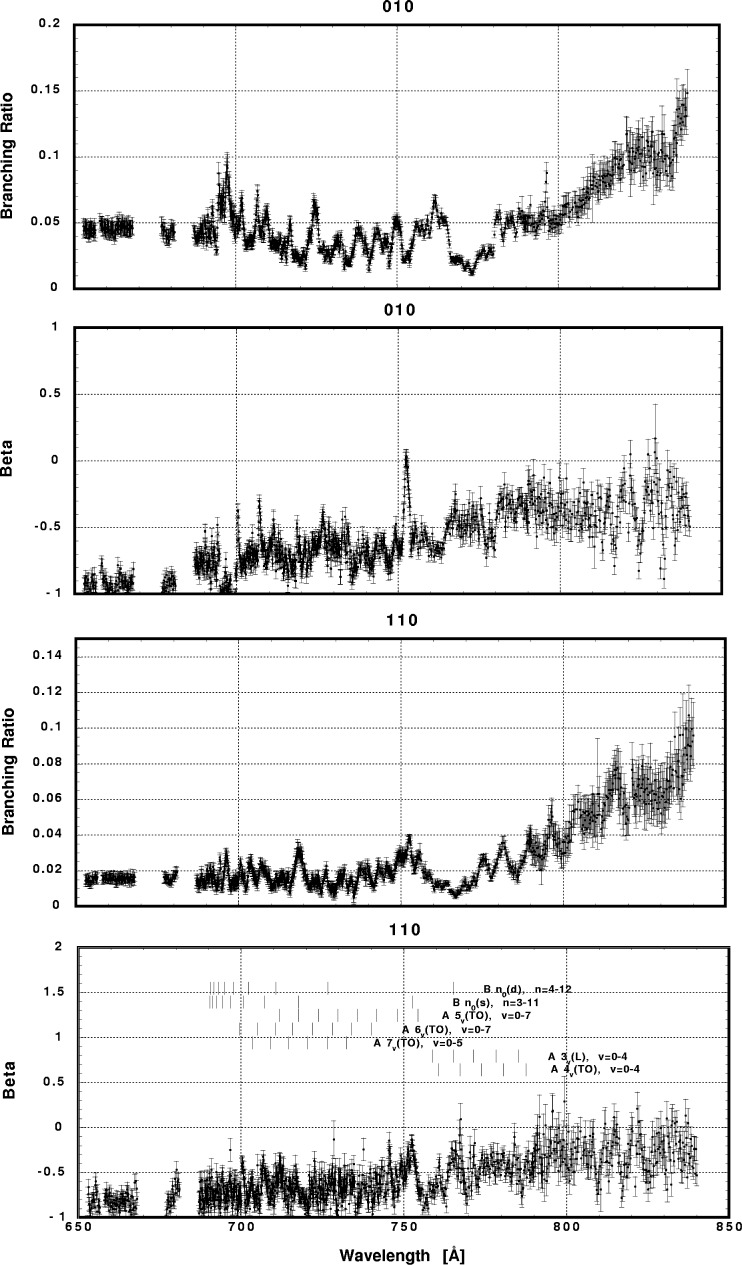
Plots of the asymmetry parameter and branching ratio for the (010) and (110) levels of the ${\text{CO}}_{2}^{+}$ ground state.

**Fig. 10:**
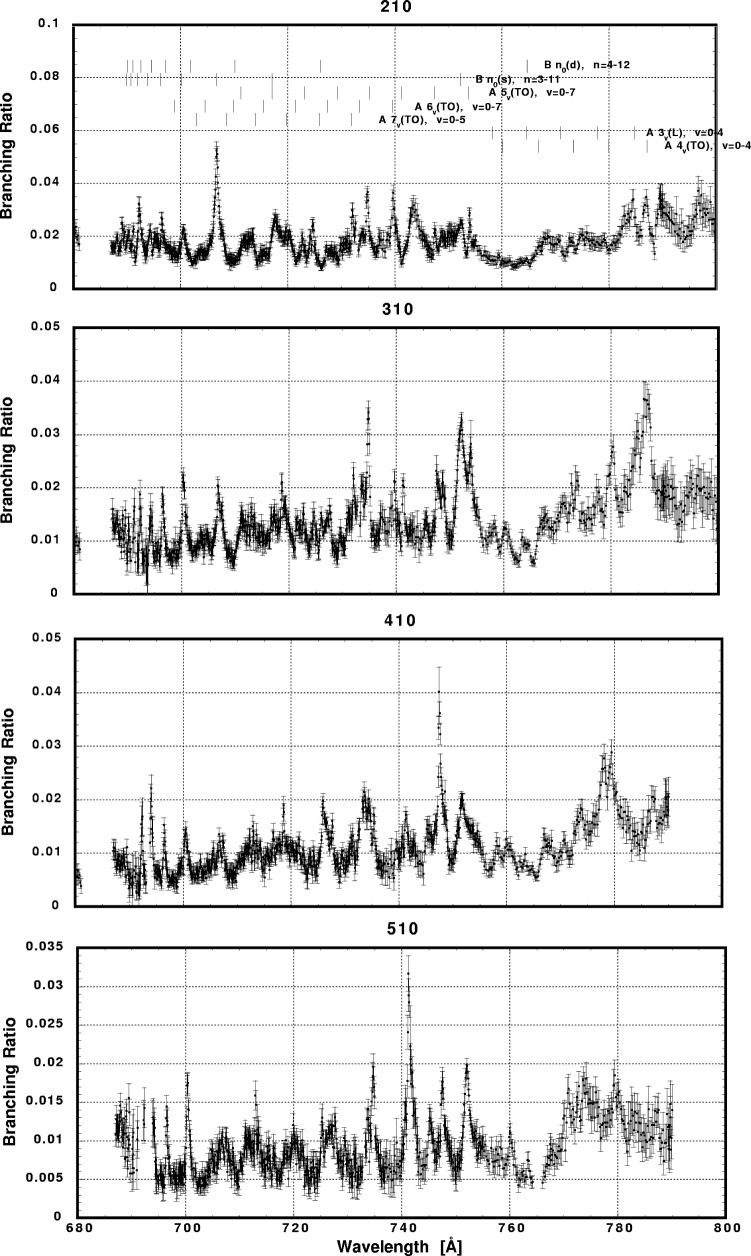
Plots of the branching ratio for the (210), (310), (410), and (510) levels of the ${\text{CO}}_{2}^{+}$ ground state.

The results presented in this work show that there is intensity in the odd bending modes, for example (*v*_1_,1,*v*_3_), contrary to the selection rules. The way in which the branching ratios and *β*-values for the bending modes respond to the resonances varies dramatically: the strong response of the (0,1,0) *β*-value to the B3_0_(s) resonance in Fig. 9, and in Fig. 10 the response of the (2,1,0) branching ratio to the B5_0_(s) resonance, the (3,1,0) branching ratio to the A5_3_(TO) resonance, the (4,1,0) branching ratio to the A5_1_(TO) resonance and the (5,1,0) branching ratio to the A5_2_(TO) resonance. However, our experiment cannot resolve these levels from the corresponding (0,0,2), (1,0,2), (2,0,2) and (3,0,2) levels, so it is not clear the selection rules are in fact being broken.

**Fig. 17:**
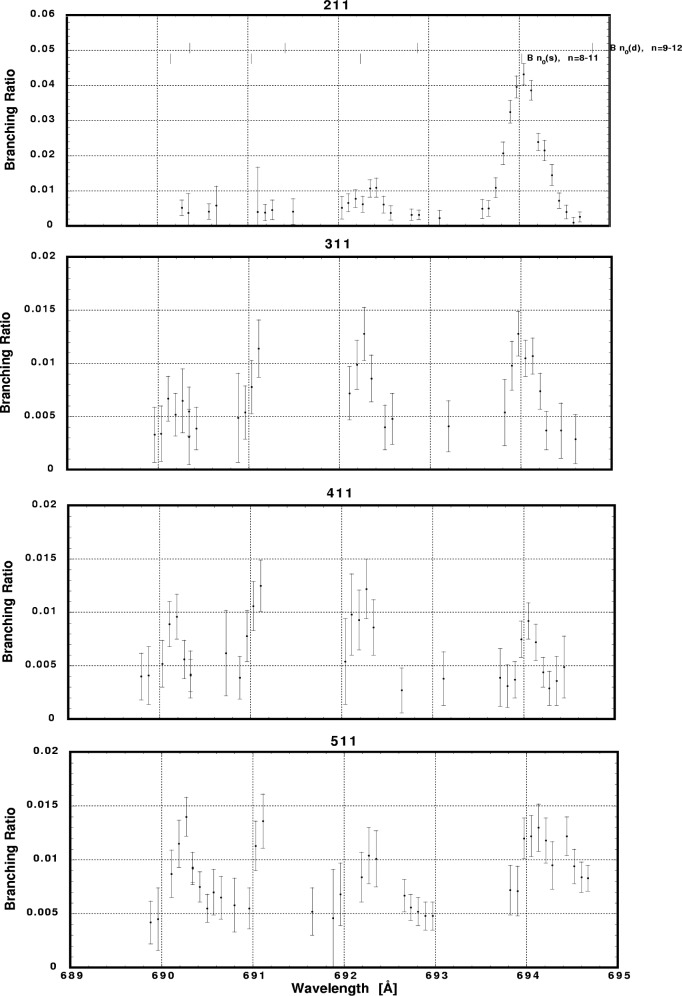
Plots of the branching ratio for the (211), (311), 411), and (511) levels of the ${\text{CO}}_{2}^{+}$ ground state.

**Fig. 18:**
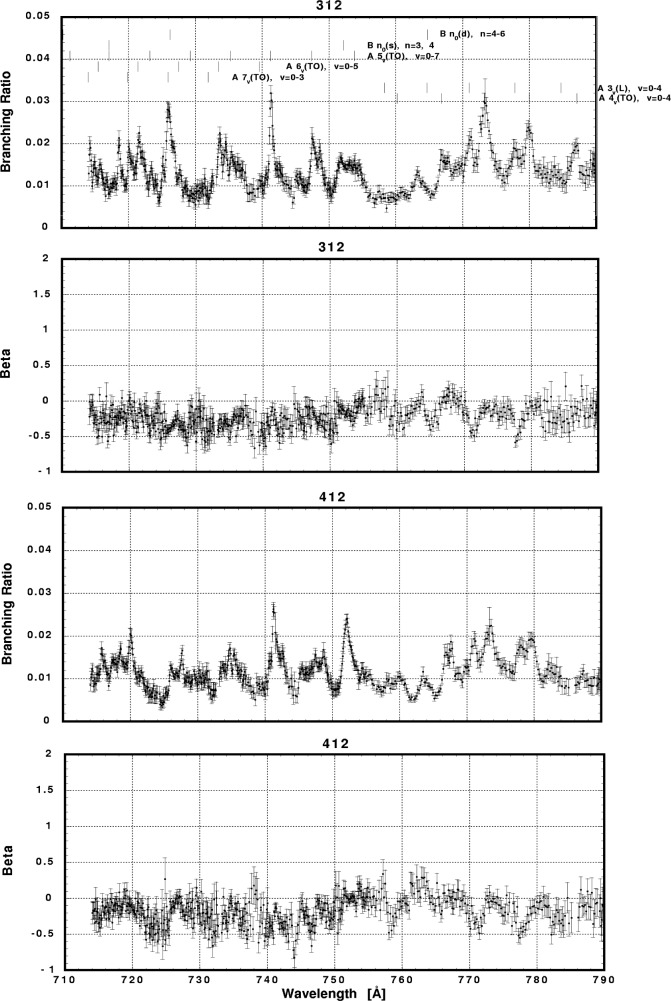
Plots of the asymmetry parameter and branching ratio for the (312) and (412) levels of the ${\text{CO}}_{2}^{+}$ ground state.

**Fig. 21:**
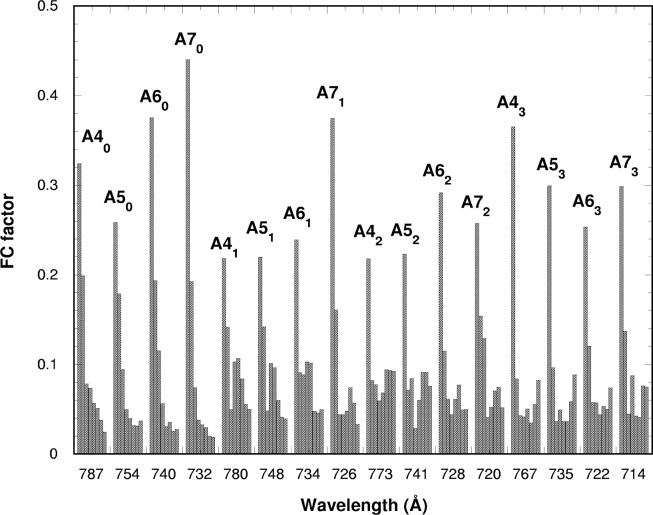
Histogram of the measured Franck-Condon factors for the ${\text{CO}}_{2}^{+}$ ground state at positions in the absorption spectrum where Rydberg autoionizing resonance levels exist. The resonances are labelled as in the text.

**Fig. 22:**
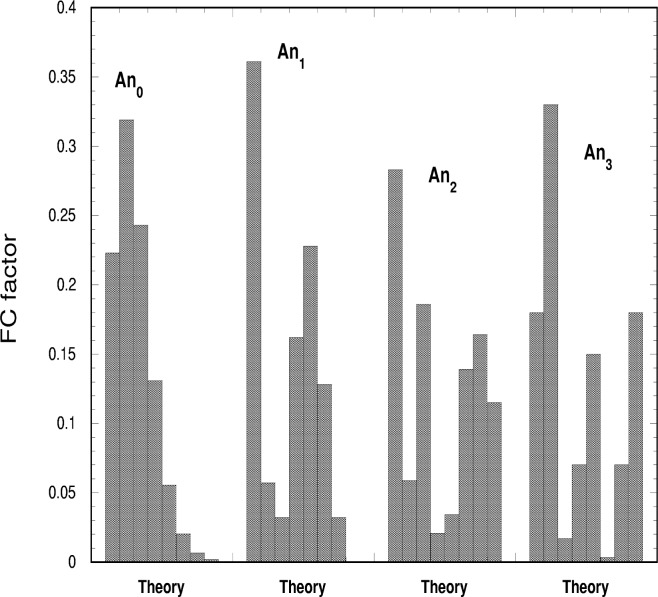
Histogram of the calculated Franck-Condon factors for the ${\text{CO}}_{2}^{+}$ ground state at positions in the absorption spectrum where Rydberg autoionizing resonance levels exist. The calculation could not distinguish between the various members of a particular series and hence the same distribution is attributed irrespective of principal quantum number. The resonances calculated are for the Tanaka-Ogawa series with vibrational levels of 0,1,2,3 quanta of the symmetric stretch in the ground electronic state of ${\text{CO}}_{2}^{+}$.

In general the response seems unpredictable; it does not appear to follow the generally accepted vibrational transition relative intensity expectations. For example, in Fig. 17 the branching ratios for the overtone modes (2,1,1) and (3,1,1) are enhanced at the B8_0_(s) resonance. The statistical errors in the corresponding values for *β* were too large for these modes to show any structure; note that the branching ratio is down at the 4 % level or less. On the other hand, in Fig. 18 where the branching ratios are down at the 2 % level, it was possible to discern some structure in the *β*-parameter measurements; the ability to do this depended very much on the quality of the fit, discussed above. However no clear pattern emerges, and it is not at all obvious what the connection is between one particular branching ratio or *β*-value being enhanced, and the resonant structure. Given the uncertainties of the identifications of the weaker vibrational levels, the conclusion must be that weak modes such as (*n*,1,0) are not necessarily enhanced by the resonances, a conclusion which is supported by the results of a higher resolution experiment which will be the subject of a forthcoming publication.

The detailed analysis of these data remains an outstanding task. The complicated nature of the interactions, and the fact that many of the resonances overlap or are incompletely resolved, makes theoretical modelling of these data very difficult. Nevertheless, one point emerges very clearly: it is necessary to make measurements of both the dynamical variables, the branching ratios and *β* parameters, in order to reveal weak structure not evident in the photoionization spectrum. The differing response of these two parameters is particularly evident in Fig. 6 for the (0,0,0) and (1,0,0) members. Furthermore, for the weaker modes, it is necessary for the experiment to have sufficient sensitivity to measure branching ratios at the 1 % level, with superior resolution to that for the data shown here, in order to be definitive.

The data described in this paper are available on the NIST web site at http://physics.nist.gov/co2arpes

## Figures and Tables

**Fig. 7:**
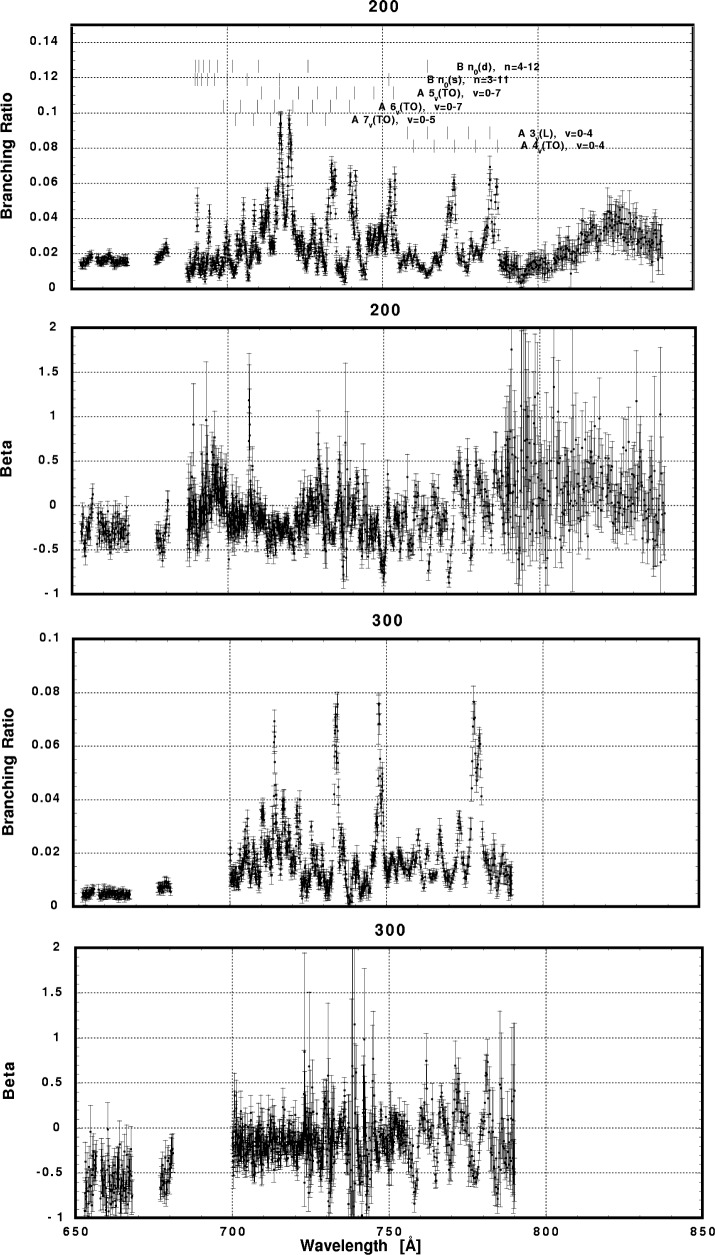
Plots of the asymmetry parameter and branching ratio for the (200) and (300) levels of the ${\text{CO}}_{2}^{+}$ ground state.

**Fig. 8:**
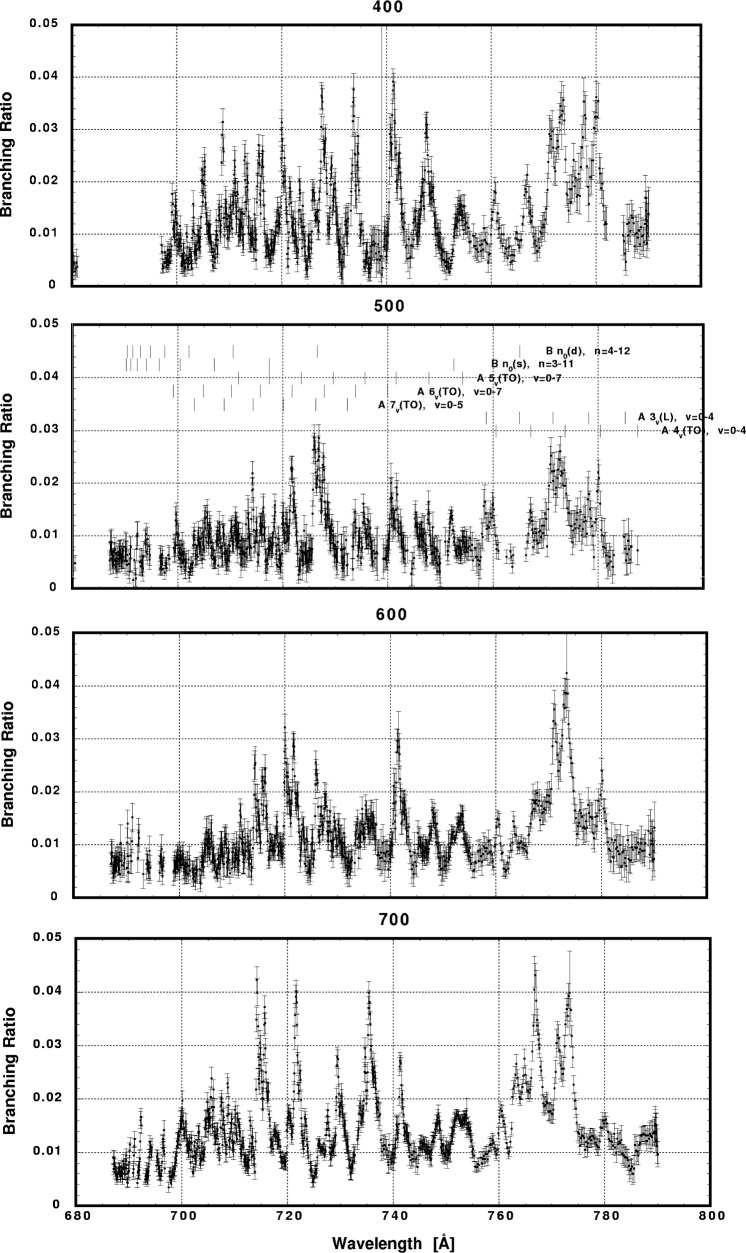
Plots of the branching ratio for the (400), (500), (600), and (700) levels of the ${\text{CO}}_{2}^{+}$ ground state.

**Fig. 11:**
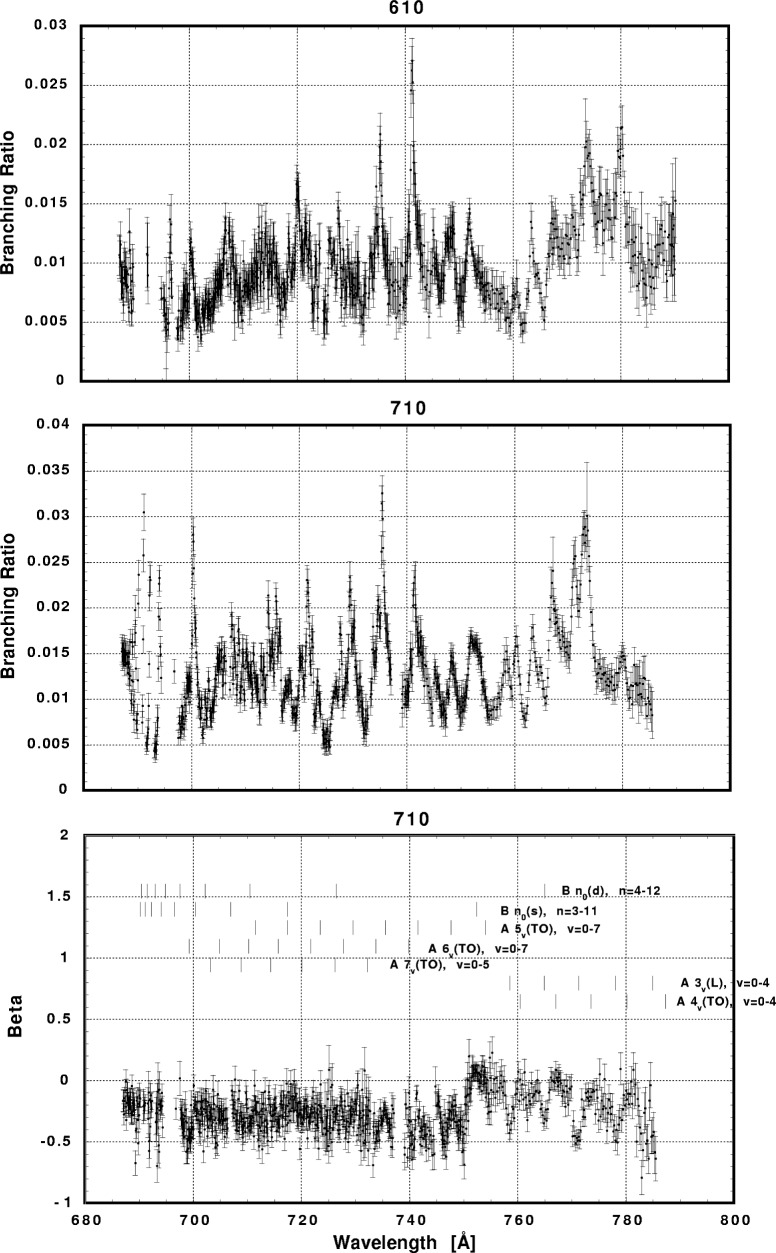
Plots of the branching ratio for the (610) and branching ratio and asymmetry parameter for the (710) levels of the ${\text{CO}}_{2}^{+}$ ground state.

**Fig. 12:**
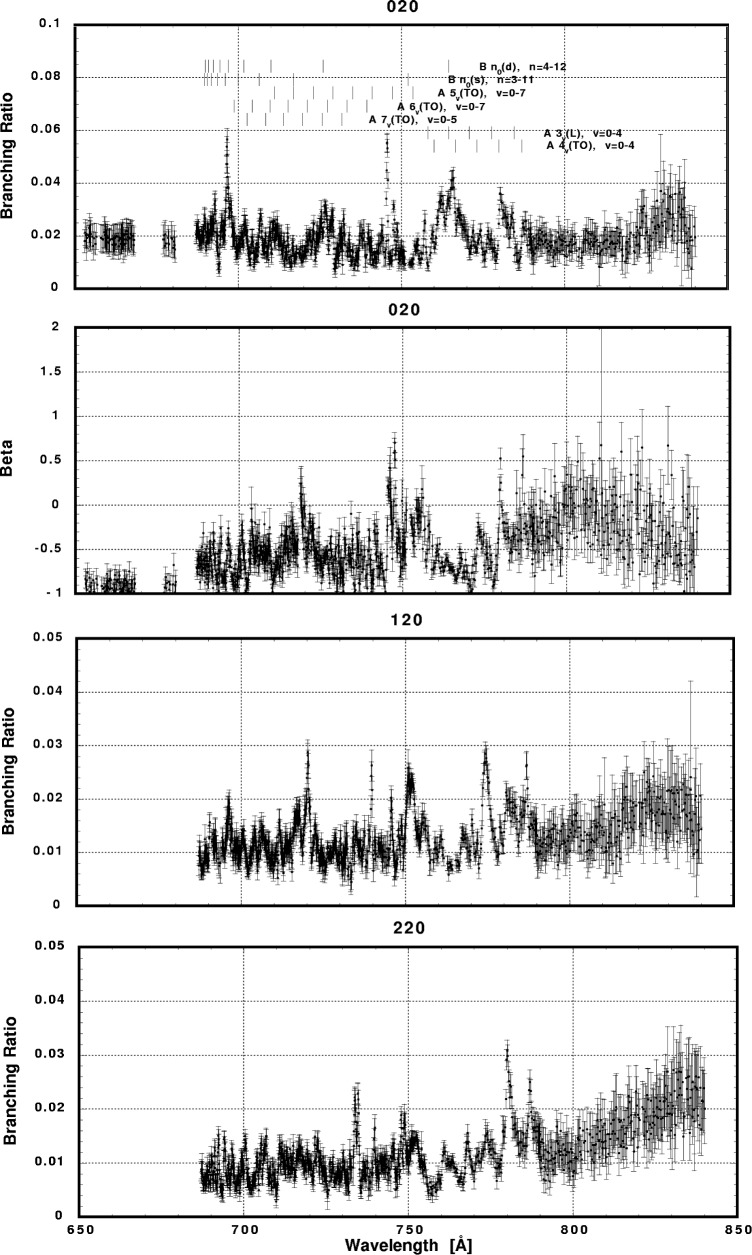
Plots of the branching ratio and asymmetry parameter for the (020) level and branching ratio for the (120) and (220) levels of the ${\text{CO}}_{2}^{+}$ ground state.

**Fig. 13:**
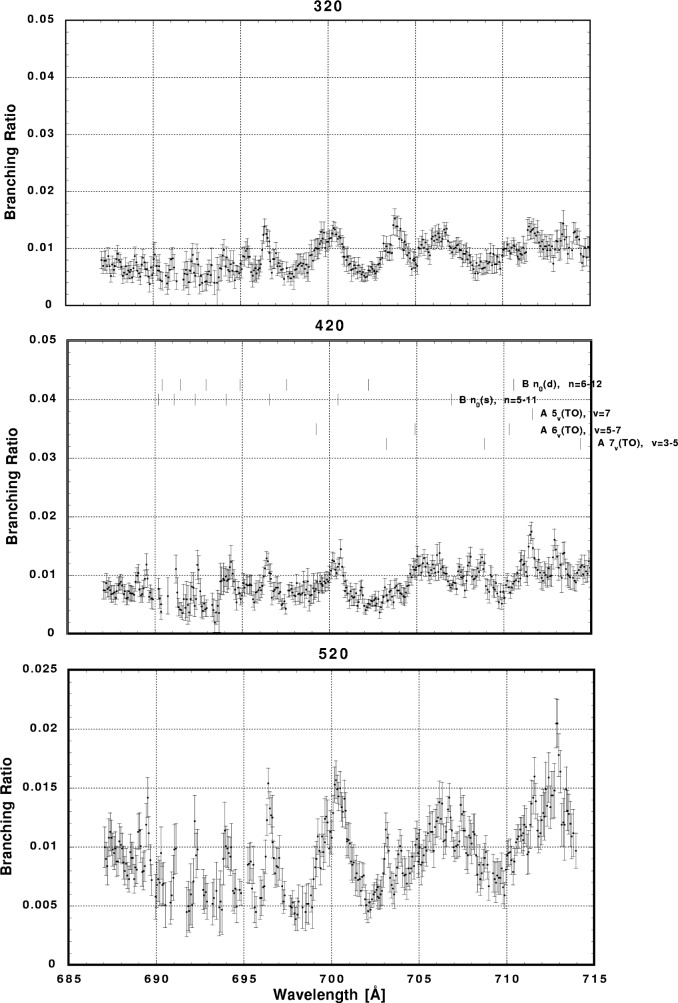
Plots of the branching ratio for the (320), (420), and (520) levels of the ${\text{CO}}_{2}^{+}$ ground state.

**Fig. 14:**
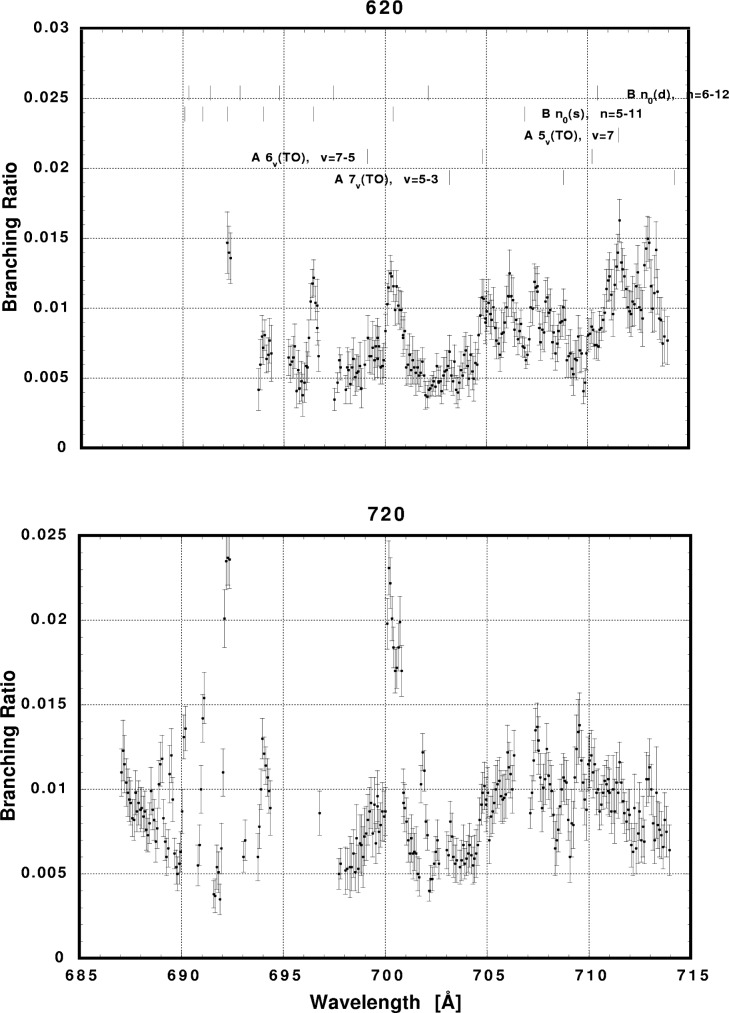
Plots of the branching ratio for the (620),and (720) levels of the ${\text{CO}}_{2}^{+}$ ground state.

**Fig. 15:**
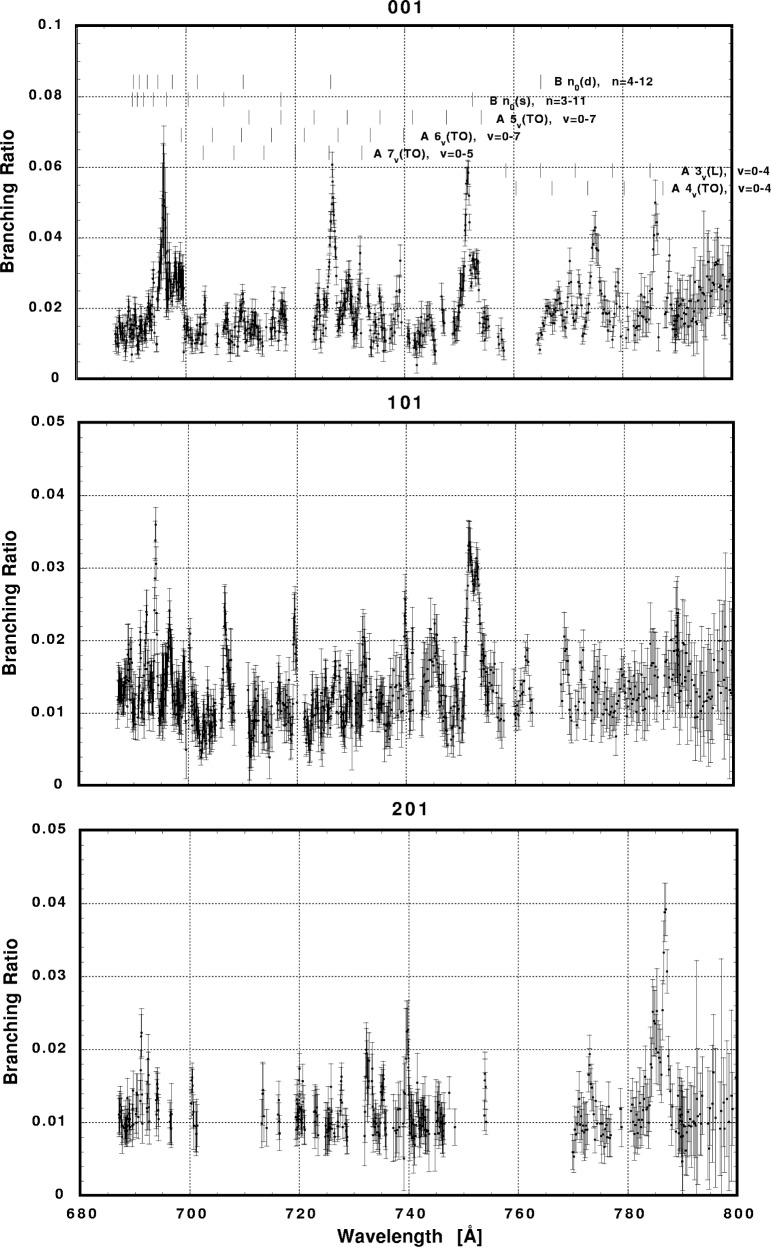
Plots of the branching ratio for the (001), (101), and (201) levels of the ${\text{CO}}_{2}^{+}$ ground state.

**Fig. 16:**
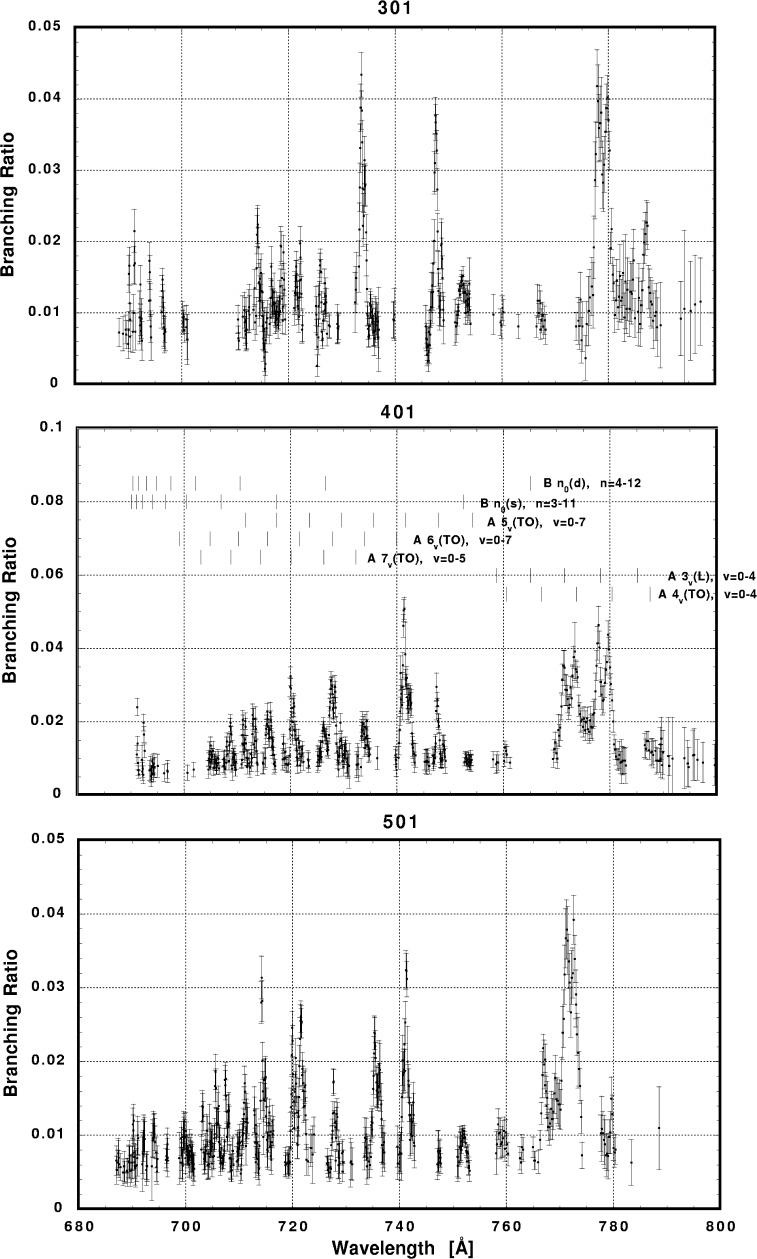
Plots of the branching ratio for the (301), (401), and (501) levels of the ${\text{CO}}_{2}^{+}$ ground state.

**Fig. 19:**
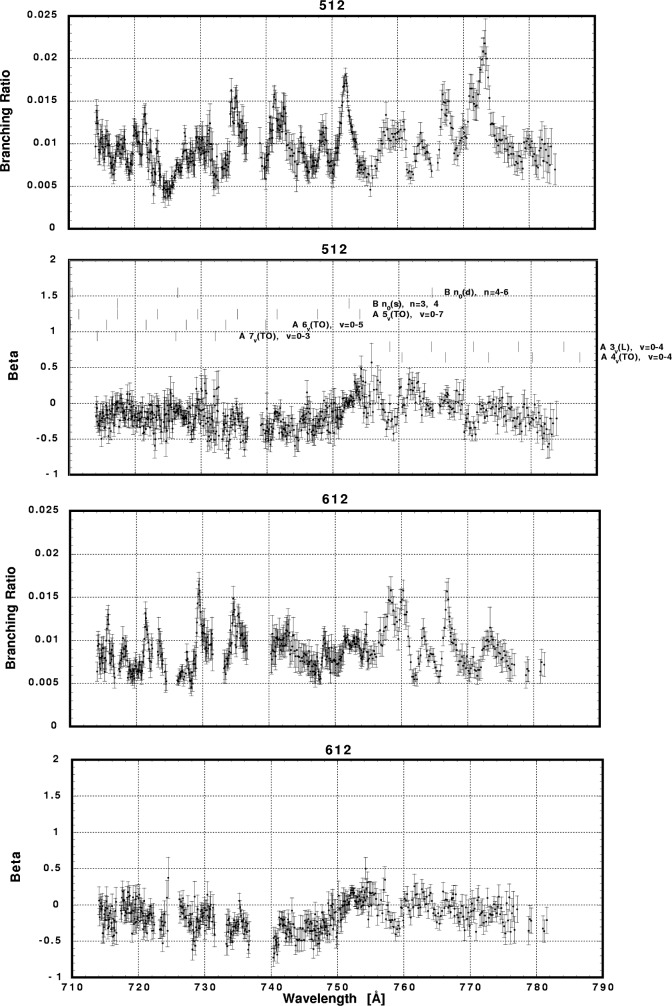
Plots of the asymmetry parameter and branching ratio for the (512) and (612) levels of the ${\text{CO}}_{2}^{+}$ ground state.

**Fig. 20:**
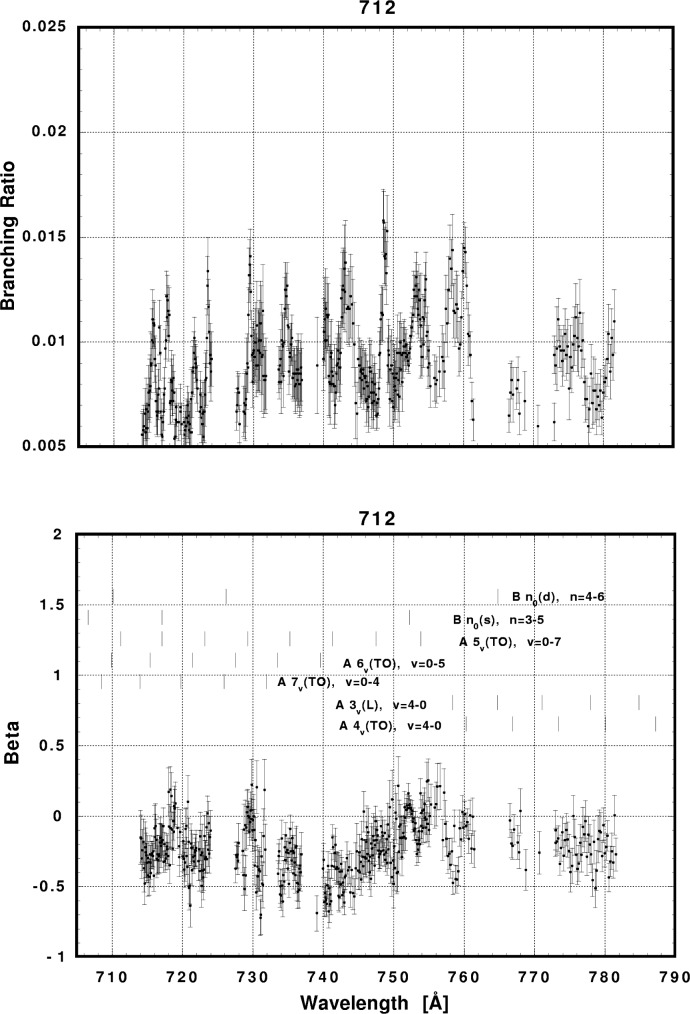
Plots of the asymmetry parameter and branching ratio for the (712) level of the ${\text{CO}}_{2}^{+}$ ground state.

**Table 1:** The vibrational levels used in the least squares fit process

Vibrational level	Energy (eV)	Vibrational level	Energy (eV)
000	0.0000	420	0.7474
010	0.0603	500	0.7835
020	0.1206	401	0.8091
100	0.1567	510	0.8438
001	0.1823	411	0.8691
110	0.2170	312	0.8944
120	0.2773	600	0.9402
200	0.3134	501	0.9658
101	0.3390	610	1.0005
210	0.3737	511	1.0261
220	0.4340	412	1.0511
300	0.4701	700	1.0969
201	0.4957	710	1.1572
310	0.5304	512	1.2078
211	0.5560	800	1.2536
320	0.5907	810	1.3139
400	0.6368	612	1.3645
301	0.6524	900	1.4103
410	0.6871	910	1.4706
311	0.7127	712	1.5212
